# Acute aortic dissection with an aberrant right subclavian artery resulting in rapid false lumen enlargement: a case report

**DOI:** 10.1186/s44215-022-00020-3

**Published:** 2023-02-06

**Authors:** Hirofumi Kasahara, Hankei Shin, Yoshito Inoue

**Affiliations:** 1 Department of Cardiovascular Surgery, Hiratsuka City Hospital, Kanagawa, Japan; 2 Department of Cardiovascular Surgery, Tokyo Dental College Ichikawa General Hospital, Chiba, Japan

**Keywords:** Aberrant right subclavian artery, Acute aortic dissection, Frozen elephant trunk

## Abstract

**Background:**

An aberrant right subclavian artery complicated by acute aortic dissection has been reported. Aneurysmal degeneration in the descending aorta adjacent to the Kommerell diverticulum in older patients has also been reported. There are concerns regarding the anatomic and pathological aspects of an aberrant right subclavian artery accompanying the Kommerell diverticulum with respect the surgical strategy for acute aortic dissection.

**Case presentation:**

We report the case of a 79-year-old man with an aberrant right subclavian artery who developed acute aortic dissection (DeBakey IIIa) and rapid enlargement of the false lumen with deteriorating dysphagia and back pain. Total arch replacement with the frozen elephant trunk technique was performed. The aberrant right subclavian artery was closed using a stent graft proximally and was ligated distally at the right side of the posterior mediastinum. To prevent injury to the esophagus, the aberrant right subclavian artery was identified by lifting the right side of the thoracic wall using a thoracotomy device for internal thoracic artery harvest to expose the dorsal circumference of the superior vena cava. Additionally, the right subclavian artery was reconstructed using an extra-anatomical bypass.

**Conclusions:**

This surgical strategy could be useful in patients with an aberrant right subclavian artery and the Kommerell diverticulum who require total arch replacement.

## Background

An aberrant right subclavian artery (ARSA) is a relatively rare abnormal development of the supra-arch vessels (prevalence of 0.5–2.0%) [1]. Surgery is recommended when concomitant dysphagia occurs either because of enlargement of the Kommerell diverticulum or compression of the esophagus or trachea where the ARSA mainly intersects from the dorsal side [2, 3]. In addition, it has been previously reported that aneurysmal degeneration in the descending aorta adjacent to the Kommerell diverticulum was frequent in older patients [4]. This case also presents these reported phenomena. An ARSA complicated by acute aortic dissection with a primary tear near the diverticulum has also been reported [5]. We report the case of a patient in whom an ARSA with aneurysmal degeneration was complicated by acute aortic dissection (DeBakey IIIa). Aortic surgery was performed because of rapid expansion of the diameter of the aorta in spite of strict blood pressure control. This case adds to the literature on the association between ARSA and arteriosclerosis and the fragility of the aortic wall. Further, this case highlights the importance of surgical planning and technique in preventing operative complications. We obtained informed consent from the patient to publish this case report.

## Case presentation

**Fig. 1:**
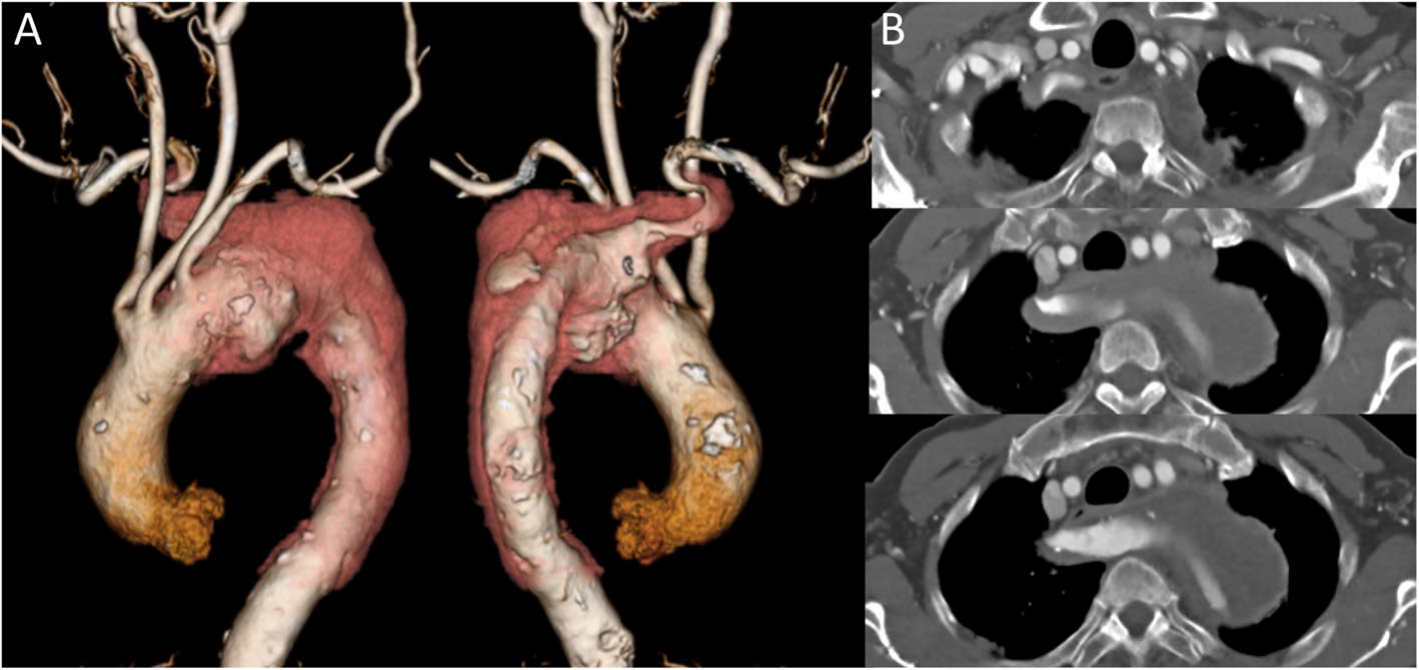
Preoperative computer tomography (CT). **A** Three-dimensional CT images (anterior and posterior). Distal aortic arch aneurysm accompanied by a Kommerell diverticulum which developed aortic dissection. The aberrant right subclavian artery (ARSA) was remarkably bent at approximately 180° twice. **B** Axial images showed that the esophagus was compressed between the trachea and the enlarged ARSA

A 79-year-old man was referred to our hospital with sudden onset of persistent back pain. Computed tomography (CT) revealed an ARSA with Kommerell diverticulum in the left aortic arch, while the distal aortic arch had aneurysmal degeneration with calcification and intraluminal thrombi. He developed acute aortic dissection (AAD) (DeBakey IIIa) with a primary tear near the Kommerell diverticulum. Antihypertensive therapy was initiated. A CT scan on the 14th day of onset revealed that the diameter of the distal arch had expanded from 54 mm to 60 mm, and the aortic dissection had progressed from the Kommerell diverticulum to the ARSA (Fig. 1). Bilateral back pain and dysphagia gradually deteriorated; therefore, urgent surgery was performed. Preoperative examinations revealed chronic renal dysfunction with an effective glomerular filtration rate of 35 mL/min and moderate to severe aortic regurgitation.

**Fig. 2:**
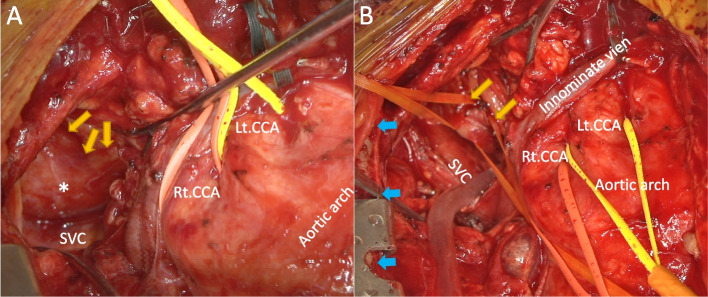
Operative findings. **A** When the superior vena cava (SVC) and the right pleura were pulled laterally and the brachiocephalic artery with the surrounding adipose tissue was pulled medially, the aberrant right subclavian artery (ARSA: the yellow arrows and asterisk) with surrounding connective tissue was visible where it was located almost posterior to the SVC. **B** The right side of the sternum (bule arrows) was lifted by the thoracotomy device for internal thoracic artery harvest. After peeling the connective tissue surrounding the ARSA, the ARSA was towed by two vascular tapes around it and pulled out anterior to the SVC. The SVC and the right pleura were retracted laterally

A median sternotomy was performed. Cardiopulmonary bypass was initiated with bicaval drainage and three arterial return sites (the left femoral artery and two 8-mm ringed expanded polytetrafluoroethylene grafts anastomosed to each of the bilateral axillary arteries), and cardiac arrest was achieved through antegrade and retrograde blood cardioplegia. Additionally, aortic valve replacement was performed using a 23-mm bioprosthesis since aortic regurgitation with a perforation in the non-coronary cusp was detected. Lower body circulatory arrest was achieved at a bladder temperature of 26 °C. Antegrade selective cerebral perfusion was initiated with the two 8-mm grafts anastomosed on the bilateral axillary arteries, and two balloon catheters were inserted into each of the bilateral common carotid arteries (CCAs). These four arterial return lines branched from one pump, and the total amount of cerebral perfusion was approximately 14 mL/kg/min. The oxygen saturation monitored using near-infrared spectroscopy was maintained at approximately 65% without any significant difference between the left and right frontal lobes. The left subclavian artery (LSA) was transected, and its proximal side was closed. Distal anastomosis in the aortic arch was performed in zone 2 with fewer aortosclerotic changes. A 150-mm-long frozen elephant trunk (FET) (J Graft Frozenix, Japan Lifeline, Tokyo, Japan) was inserted to ensure that the primary tear and the Kommerell diverticulum were covered. A 35-mm stent graft was selected, with a diameter 110% larger than the diameter of the descending aorta, where the distal portion of the FET was going to be deployed. The ARSA was ligated behind the superior vena cava (SVC) to prevent injury to the esophagus and was ligated at two sites to prevent recanalization in the chronic phase (Figs. 1 and 2). We identified the ARSA in advance; since the ARSA ran deep in the mediastinum, before heparinization, lifting the right side of the thoracic wall using a thoracotomy device for internal thoracic artery harvest was useful to expose the dorsal circumference of the SVC (Fig. 2).

During distal anastomosis of the aortic arch, the inside of the FET was occluded with a balloon, and lower body perfusion (flow rate 500–700 mL/min and blood temperature of 25 °C) was resumed via the femoral artery cannula. The lower body circulatory arrest lasted 28 min. After anastomosing the FET with a four-branched graft (J Graft, Japan Lifeline, Tokyo, Japan), antegrade systemic perfusion was resumed from the side branch of the prosthesis. The LSA and left CCA were individually reconstructed using the side branches. A proximal aortic anastomosis was performed, the aortic clamp was released, and the right CCA was reconstructed. Finally, a pre-anastomosed right axillary artery graft was guided inside the anterior mediastinum and was anastomosed with the side branch (right subclavian artery reconstruction). The myocardial ischemia duration was 168 min, and the total cardiopulmonary bypass duration was 211 min.

**Fig. 3:**
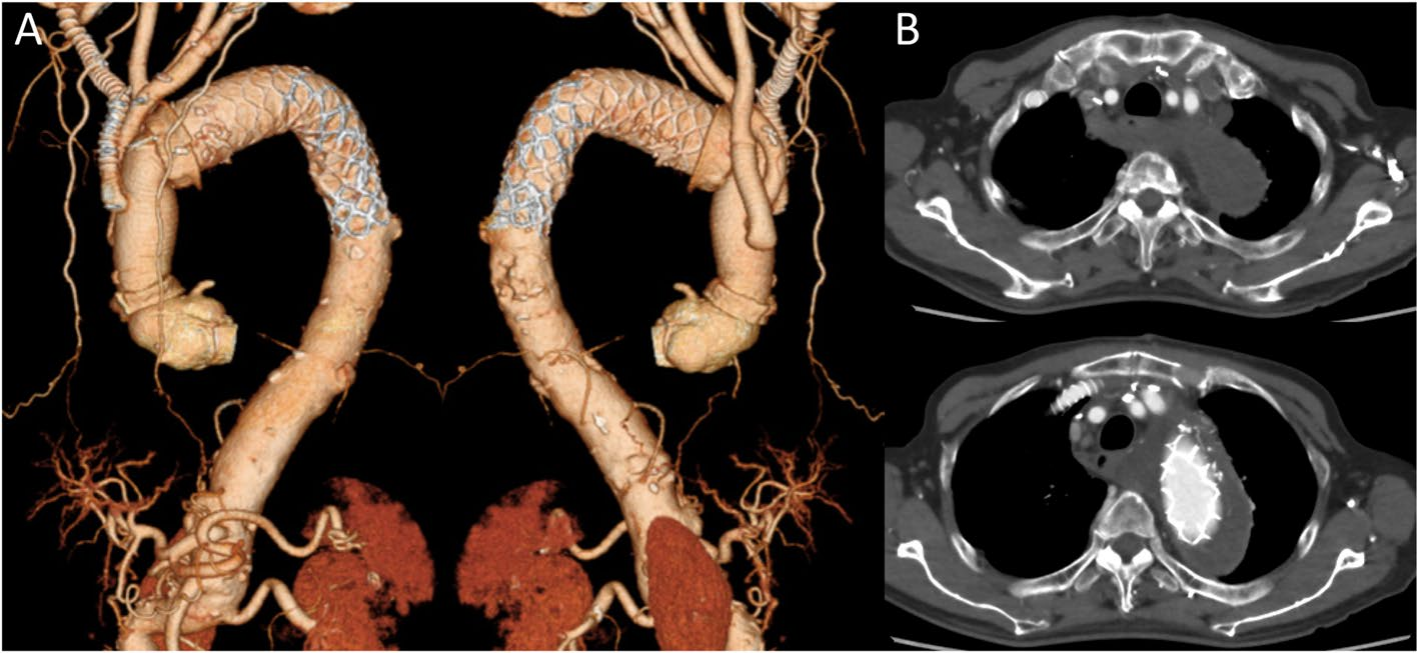
Postoperative computer tomography (CT). **A** Three-dimensional CT images (anterior and posterior). The right subclavian artery was reconstructed using a ringed expanded polytetrafluoroethylene graft. **B** Axial images showed that the aberrant right subclavian artery and the distal arch aneurysm were completely thrombosed

The patient was extubated the day after surgery. His chest symptoms disappeared promptly, and his dysphagia also improved. The postoperative course was uneventful, and he was discharged without any complications. A CT scan before discharge revealed that no blood flow in the pseudo lumen, and the ARSA was completely thrombosed (Fig. 3). The CT scan images captured 1 year after surgery revealed further shrinkage of the aneurysm.

## Discussion

Surgical treatment for the Kommerell diverticulum can be broadly divided into open surgery and thoracic endovascular aortic repair [6]. In open surgery, the approach is determined by the extent of replacement and the ARSA reconstruction method. Surgery can be performed with a lateral thoracotomy if concomitant graft replacement from the ascending to the aortic arch branches is not required [3]. We performed surgery with a median sternotomy because of the patient’s aortic regurgitation, distal arch aneurysm, and our policy regarding antegrade ARSA reconstruction.

However, there are concerns regarding the anatomic and pathological aspects of an ARSA. Unlike normal patients, the right axillary artery cannot perfuse the right carotid artery. Additionally, a high frequency of aneurysmal and medial degeneration in the Kommerell diverticulum has been reported [4]. Kim et al. performed open aortic repair in 19 patients with a mean age of 48 years. Pathologic specimens of the Kommerell diverticulum and aorta were examined in 18 patients, and medial degeneration was noted in all patients [7]. Additionally, aneurysmal degeneration with a thick intraluminal thrombus in the distal aortic arch was observed in our patient. We prefer to perfuse cerebral blood flow at healthy vessels without atherosclerosis. Therefore, we used four arterial return lines for cerebral perfusion, including the left axillary artery.

To prevent spinal cord injuries associated with the FET technique, the femoral artery was perfused during the distal anastomosis not only for flushing debris and air, but also for deploying the FET device after filling of the blood in the descending aorta. To shorten the ischemic duration, lower body circulation was resumed within 30 min using a balloon occlusion technique inside the FET. The distal end of the stent graft should be deployed above T9. The absence of LSA perfusion was also reported as a risk factor for spinal cord injury [8]. We have addressed these concerns above.

The vessel diameter of the ARSA expanded owing to the progression of the AAD to the ARSA itself, and the trachea and upper esophagus were further compressed. The orifice of the ARSA and Kommerell diverticulum could be occluded by a stent graft; however, esophageal decompression may be insufficient. It was necessary to completely thrombose the ARSA and Kommerell diverticulum by ligating the ARSA in the posterior mediastinum to relieve the esophagus, as without adventitial tissue, it is vulnerable to the surrounding pressure. To prevent esophageal injury during surgery, we dissected and mobilized the ARSA to ligate it away as far as possible from the esophagus. Based on preoperative CT findings (Figs. 2 and 3), it was almost behind the SVC and at the right-site of the posterior mediastinum. Transesophageal echocardiography was avoided because there was considerable pressure on the esophagus from the ARSA with aortic dissection. A nasogastric tube was carefully inserted, and the location of the esophagus was identified by palpating the tube from the outside. The ARSA was identified easily by lifting the right side of the thoracic wall using a thoracotomy device for internal thoracic artery harvest during coronary artery bypass grafting to expose the dorsal circumference of the SVC (Figs. 1 and 2). Since the ARSA was remarkably bent (approximately 180° twice; Fig. 1), its appearance, including that of the surrounding connective tissue before dissection, was semicircular and had a unique shape (Fig. 2A). In order to omit taping the ARSA proximal site, coil-embolization could have been used. However, our method is feasible and convenient, without using an intraoperative X-ray fluoroscope. In addition, intraoperative findings showed a certain amount of backflow via the ARSA. We believe that it is desirable to surgically and reliably ligate the ARSA to prevent type II endoleaks, maintain intraoperative cerebral perfusion, and ensure a bloodless operative field.

ARSA reconstruction methods include in-site reconstruction, non-anatomical bypass (including right common carotid artery-axillary artery anastomosis by cervical incision), or not performing reconstruction (ligation only). Esposito et al. reported severe upper limb ischemia in two of nine patients without reconstruction [9]. In our patient, the AAD extended from the Kommerell diverticulum to the ARSA; therefore, we preferred to anastomose at the axillary artery from the four-branched graft to the healthy distal vessels. There are reports of in-site reconstruction [3, 5], but in our patient, the ARSA was large and highly meandering, the site of anastomosis was deep, and a proper graft route was not easy to determine as it traveled dorsally. Using a small-diameter prosthesis pre-anastomosed to the axillary artery seems to be a safe and useful method for reconstruction with antegrade flow.

In some patients, surgical removal of the Kommerell diverticulum or vascular ring division may be necessary to improve subjective symptoms such as dysphagia [6]. Although our method left the ARSA on the dorsal side of the esophagus, the aneurysmal sac of the Kommerell diverticulum did not directly compress the esophagus, and the patient’s subjective symptoms improved immediately post-surgery. The reduction of the ligated vessel diameter was not remarkable immediately after the surgery; however, we speculate that back pain and dysphagia were improved by decompression due to complete thrombosis of the ARSA with loss of arterial pressure, and by loosened contact with the esophagus due to complete dissection of the ARSA for ligation.

Although these methods have been previously presented, the use of four arterial return lines to prevent cerebral infarction, the shortening of the ischemic duration of the lower body circulatory arrest period to prevent spinal cord ischemia, and ligation of the ARSA under direct vision can provide new information for the management of ARSA.

## Conclusion

We presented a case of a patient with a Kommerell diverticulum with aneurysmal degeneration who developed acute aortic dissection with a primary tear near the diverticulum. A median sternotomy approach using the FET technique ensured a one-time radical surgery with reliable cerebral protection by multiple arterial return lines. An ARSA should be ligated in the posterior mediastinum. Lifting the right thorax by a thoracotomy device made the procedure easier and safer by avoiding esophageal injury and exposing the dorsal circumference of the SVC.

## Data Availability

The datasets used and/or analyzed during the current study are available from the corresponding author on reasonable request.

## Abbreviations

AAD: Acute aortic dissection
ARSA: Aberrant right subclavian artery
CCA: Common carotid artery
CT: Computed tomography
FET: Frozen elephant trunk
LSA: Left subclavian artery
SVC: Superior vena cava
